# Matrix Mechanotransduction via Yes-Associated Protein in Human Lamina Cribrosa Cells in Glaucoma

**DOI:** 10.1167/iovs.63.1.16

**Published:** 2022-01-11

**Authors:** Rory Murphy, Mustapha Irnaten, Alan Hopkins, Jeffrey O'Callaghan, W. Daniel Stamer, Abbot F. Clark, Deborah Wallace, Colm J. O'Brien

**Affiliations:** 1 Department of Ophthalmology, Mater Misericordiae University Hospital, Dublin, Ireland; 2 Clinical Research Centre, School of Medicine, University College Dublin, Dublin, Ireland; 3 Ocular Genetics Unit, Smurfit Institute of Genetics, University of Dublin, Trinity College, Dublin, Ireland; 4 Duke Eye Centre, Durham, North Carolina, United States; 5 Department of Cell Biology & Immunology and the North Texas Eye Research Institute, U. North Texas Health Science Centre, Ft. Worth, Texas, United States

**Keywords:** lamina cribrosa, YAP, VERTEPORFIN, glaucoma, STIFFNESS

## Abstract

**Purpose:**

Extracellular matrix stiffening is characteristic of both aging and glaucoma, and acts as a promoter and perpetuator of pathological fibrotic remodeling. Here, we investigate the role of a mechanosensitive transcriptional coactivator, Yes-associated protein (YAP), a downstream effector of multiple signaling pathways, in lamina cribrosa (LC) cell activation to a profibrotic, glaucomatous state.

**Methods:**

LC cells isolated from glaucomatous human donor eyes (GLC; n = 3) were compared to LC cells from age-matched nonglaucomatous controls (NLC; n = 3) to determine differential YAP expression, protein levels, and proliferation rates. NLC cells were then cultured on soft (4 kPa), and stiff (100 kPa), collagen-1 coated polyacrylamide hydrogel substrates. Quantitative real-time RT-PCR, immunoblotting, and immunofluorescence microscopy were used to measure the expression, activity, and subcellular location of YAP and its downstream targets, respectively. Proliferation rates were examined in NLC and GLC cells by methyl thiazolyl tetrazolium salt assays, across a range of incrementally increased substrate stiffness. Endpoints were examined in the presence or absence of a YAP inhibitor, verteporfin (2 µM).

**Results:**

GLC cells show significantly (*P* < 0.05) increased YAP gene expression and total-YAP protein compared to NLC cells, with significantly increased proliferation. YAP regulation is mechanosensitive, because NLC cells cultured on pathomimetic, stiff substrates (100 kPa) show significantly upregulated YAP gene and protein expression, increased YAP phosphorylation at tyrosine 357, reduced YAP phosphorylation at serine 127, increased nuclear pooling, and increased transcriptional target, connective tissue growth factor. Accordingly, myofibroblastic markers, α-smooth muscle actin (α-SMA) and collagen type I, alpha 1 (Col1A1) are increased. Proliferation rates are elevated on 50 kPa substrates and tissue culture plastic. Verteporfin treatment significantly inhibits YAP-mediated cellular activation and proliferation despite a stiffened microenvironment.

**Conclusions:**

These data demonstrate how YAP plays a pivotal role in LC cells adopting a profibrotic and proliferative phenotype in response to the stiffened LC present in aging and glaucoma. YAP provides an attractive and novel therapeutic target, and its inhibition via verteporfin warrants further clinical investigation.

Glaucoma, a predominantly age-related, progressive optic neuropathy, is the leading cause of irreversible blindness worldwide. Compounded by an aging population, its prevalence is projected to increase from 76 to 112 million between 2020 and 2040.^1^ This will have profound socioeconomic ramifications, heralding an urgent need to investigate novel therapeutic targets. Despite advances in current treatment modalities for glaucoma, there is universal reliance on reducing elevated intraocular pressure (IOP), the primary risk factor for POAG. Unfortunately for many individuals, IOP lowering proves insufficient to slow pathological remodeling of the lamina cribrosa's (LC) extracellular matrix (ECM), and subsequent loss of vision.^2^^,^^3^ Alternatives, such as directly targeting the underlying remodeling at the LC seems to be a particularly attractive yet elusive option. However, therapeutically targeting the LC requires further insights into the molecular mechanisms culpable, which we sought to explore in this study.

Variation in both susceptibility and progression of glaucomatous optic neuropathy across a range of IOP is intriguing and may in part be explained by biomechanics differences at the optic nerve head. The LC is a fenestrated, meshlike structure across the optic nerve head, composed of collagenous load-bearing beams that support vulnerable, unmyelinated retinal ganglion cell axons as they exit the eye posteriorly forming the optic nerve.^4^^,^^5^ The LC is subject to a dynamic, continuous set of forces, with resultant cyclic stress and strain acting across it. It endures IOP forces at the anterior face and cerebrospinal fluid pressure forces at the posterior face, forming the suitably named translaminar pressure gradient, which itself is implicated in the pathogenesis of glaucoma.^6^ The ability to resist deformation from these forces is partly determined by physical properties of the LC (stiffness), which increase with age (through age-related glycation cross-linking of the fibrillar ECM), race and persistently raised IOP, resulting in a less compliant, remodeled LC and more severe corresponding visual field defects.^7^^–^^12^

The resident cells of the LC respond to extracellular mechanical cues via mechanotransduction.^13^ The LC is populated by astrocytes that stain positive for glial fibrillary acidic protein (GFAP), and GFAP-negative, fibroblast-like cells termed “LC cells,” fundamental for the maintenance of the surrounding ECM.^14^^,^^15^ LC cells constitutively express α-SMA, elastin, COL1A1, and fibronectin, and previous work by our group has shown that LC cells respond to mechanical stretch, TGF-β1, hypoxia, and oxidative stress, upregulating expression of well-known ECM genes in a profibrotic manner.^16^^–^^21^ Furthermore, we have recently shown that increased substrate stiffness alone elicits an activated myofibroblastic, glaucomatous phenotype from previously quiescent nonglaucomatous LC cells.^22^ ECM stiffness with both quantitative and qualitive remodeling is a hallmark of chronic fibrotic disease; prevalent in most systemic and ocular pathologies.^23^ We hypothesize that the biomechanical stiffness of the LC, as perceived by its resident cells, acts as a cause and is a consequence of fibrosis and further stiffening in glaucoma in a positive feedback manner.^24^

To disrupt this maladaptive positive feedback loop, we targeted the potent, mechanosensitive, transcriptional coactivator, Yes-associated protein (YAP), a key player in multiple mechanotransduction pathways, and main downstream effector of the Hippo pathway.^25^ YAP is a transcriptional coactivator that associates with DNA via its interaction with TEA domain (TEAD) DNA-binding proteins to induce transcription of target genes.^26^ Its transcriptional activity regulates key biologic functions including proliferation, differentiation, and apoptosis and is heavily involved in mechanosensitive upstream signaling pathways including F-actin/Caveolin-1/Rho, Hippo, TGF-β/Smad, PI3K/MAPK, Wingless/Int (Wnt), and, to a degree, cellular bioenergetics.^27^^–^^30^

There are many upstream regulators of YAP activity, which include mechanical cues (cell to cell adhesion, cell polarity, cell density, tissue architecture, shear stress, and ECM stiffness), extracellular ligands (lysophosphatidic acid [LPA], sphingosine 1-phosphate, and epidermal growth factor), which signal via G protein coupled receptors (GPCR) and receptor tyrosine kinases, inflammation (through TNF-α), hypoxic stress (through SIAH2 ubiquitin E3), and energy stress (Hippo pathway via AMP-activated protein kinase), with mechanical cues setting an overarching responsiveness to GPCR, Wnt, and Hippo signaling pathways.^31^^–^^33^ The mechanosensing ability of the cell is largely achieved via focal adhesions and subsequent actin-cytoskeleton remodeling.^34^

The transcriptional activity of YAP predominantly depends on its phosphorylation status. More specifically, the site of phosphorylation subsequently determines its subcellular location. For example, large tumor suppressor (LATS1/2)–mediated YAP phosphorylation at serine 127 (pYAP[s127]) (via the Hippo pathway) leads to direct binding of protein 14-3-3, which favors cytosolic retention through increased nuclear export, thus limiting its transcriptional ability. Whereas YAP phosphorylation at tyrosine 357 (pYAP[y357]) (via SRC family kinases) promotes nuclear pooling, subsequently upregulating its transcriptional activity of target genes through binding to TEAD transcription factors.^28^^,^^35^^–^^37^ Nuclear accumulation and upregulation of YAP has been implicated in the pathogenesis of multiple cancers, in addition to multisystem fibrotic diseases.^38^ In vitro, cancer-associated fibroblasts subjected to a stiffened microenvironment demonstrate YAP nuclear translocation, transcription of profibrotic YAP target genes, and increased proliferation.^39^

In this study, we aim to expand our mechanistic understanding of the role of YAP in glaucoma by studying LC cells from nonglaucomatous and glaucomatous eye donors. Furthermore, we interrogate the effect of exposing healthy, nonglaucomatous LC cells to a pathomimetic, stiffened in vitro microenvironment, akin to the glaucomatous milieu. Finally, we use a recently described YAP inhibitor, verteporfin (VP), in an attempt to disrupt LC mechanotransduction, precluding any subsequent profibrotic, positive-feedback cycle.

## Methods

### LC Cell Culture and Characterization

Human LC cells (supplied by Alcon Labs, Fort Worth, TX, USA; Duke University, Durham, NC, USA) were isolated and cultured after retrieval from donors as previously described.^16^ All experiments were performed in compliance with the tenets of the Declaration of Helsinki. Cells were characterized for markers such as α-SMA while being negative for GFAP (an astrocyte marker) and ionized Ca^2+^ binding adapter molecule 1 (a microglial marker).^16^^,^^40^ Human LC cells were used from age-matched healthy donor eyes with no history of glaucoma, ocular disease, or other neurologic diseases (n = 3) and also from donors with physician-confirmed glaucoma (n = 3). For experiments comparing NLC to GLC cells, the mean age of nonglaucomatous donors was 73 ± 6.6 years (67, 72, 80) and 75 ± 7.5 years for glaucomatous donors (68, 74, 83). For the experiments using NLC cells on altered substrate stiffness and YAP inhibition with VP, we used LC cells from nonglaucomatous donors aged 26, 40, and 61 years. When required, frozen cells were removed from a liquid nitrogen cryostore and rapidly (<1 minute) transferred to a water bath prewarmed at 37°C. The freshly thawed cells were slowly added to prewarmed growth medium and plated at high density to optimize recovery under a laminar flow hood using aseptic technique before their culture under standard conditions. LC cells were cultured at 37°C with 95% humidified air atmosphere and 5% CO_2_ in Dulbecco's modified Eagle medium containing 10% (vol/vol) fetal bovine serum and 1% L-glutamine, and 1% penicillin-streptomycin. Cells were cultured in full media until 80% to 90% confluence was attained and passaged using trypsin/ EDTA for one to two minutes at 37°C. The cells were spun in a centrifuge at 1200*g* for five minutes, and the supernatant was aspirated and discarded. The pellet was then resuspended in fresh media for subsequent culturing of cells. For all experiments passages 4 to 8 were used, and for comparative experiments, cells from equal passage were used. Unless otherwise stated, reagents and solutions were from Sigma-Aldrich Corp. (St. Louis, MO, USA).

### LC Culture on Stiffened Matrices

LC cells were seeded at a low-density of 2000 cells/cm^2^ on commercially available collagen-1 (rat tail)–coated polyacrylamide hydrogel substrates (Softwell, Matrigen Products; Cell Guidance Systems, Cambridge, UK) of substrate stiffness 4 kPa and 100 kPa. The differential expression of YAP in LC cells grown under these controlled stiffness conditions was examined by Western immunoblotting, qRT-PCR and immunofluorescence. For immunofluorescence, 0.17 mm × 30 mm coverslips placed under the collagen-1–coated polyacrylamide hydrogel substrates (Softslip, Matrigen Products; Cell Guidance Systems) were used. Cell culture on these collagen-coated coverslips enabled easier removal for image capture.

### Verteporfin Treatment

Dimethyl sulfoxide (DMSO) was used to reconstitute VP (SML0534; Sigma-Aldrich) to stock solutions of 2 or 4 µM. NLC cells were seeded at a density of 2000 cells/cm^2^ onto substrates for 24 hours, serum-restricted pretreatment for 12 hours, and then subjected to either vehicle only DMSO (control), or VP treatment (VP/DMSO) for a further 24 hours, at which point they were immediately isolated and analyzed. The altered gene expression levels, protein expression, and subcellular location of YAP were assessed as: Untreated control (4 kPa substrate) and treated (100 kPa substrate) groups. Both groups were examined in the absence or presence of VP. A minimum of three independent experiments from different LC cell donors were performed. The duration of culture was intended to be short enough to avoid any potential differences in proliferation confounding results, as cell-cell contact is a negative regulator of YAP and its nuclear translocation and could diminish or reverse any findings specific to substrate stiffness. VP use in the proliferation/ methyl thiazolyl tetrazolium salt (MTS) assay was at a concentration of 2 and 4 µM for a period of 24 hours after a 24-hour period for cell attachment and overnight serum starve in serum-free medium.

### Protein Extraction and Western Immunoblotting

On completion of treatments, media was removed and cells incubated in 1X ice-cold PBS. The cells were then trypsinized into ice-cold PBS, spun in a centrifuge at 14,000*g* for five minutes at 4°C to form a pellet. The supernatant was carefully removed without disturbing the pellet and discarded. Crude cell lysate was collected using radio immuno-precipitation assay buffer containing protease inhibitor cocktail, and then the cells were incubated on ice for five minutes and subsequently spun in a centrifuge for 15 minutes at 15,000*g* at 4°C. The cleared supernatant was collected, and the protein concentration was quantified using the Bradford method. Protein extracts (20 µg) were electrophoresed on 10% polyacrylamide-SDS gels and transferred to a nitrocellulose membrane. For control and treated samples on soft and stiff substrates, equal amounts of protein were loaded in the SDS-PAGE. Membranes were blocked with 5% non-fat milk in Tris-buffered saline solution containing 0.1% Tween-20 at room temperature for one hour before incubation overnight at 4°C with primary antibodies as outlined in Table 1. After being washed three times in Tris-buffered saline solution containing 0.1% Tween-20, membranes were incubated for one hour at room temperature with appropriate secondary antibodies including goat anti-rabbit IgG-horseradish peroxidase–conjugated secondary antibody (Cell Signaling Technology, Danvers, MA, USA) and mouse IgG kappa binding protein (m-IgGk BP) conjugated to horseradish peroxide (Santa Cruz Biotechnology, Dallas, TX, USA). Membranes were reprobed with anti-β-actin (Cell Signaling Technology) as loading controls. The blots were then processed according to standard protocols using enhanced chemiluminescence detection reagents (Fisher Scientific, Waltham, MA, USA), the LI-COR C-DiGit blot scanner (LI-COR Biosciences, Lincoln, NE, USA) and densitometry analysis via I Image Studio software (LI-COR Biosciences) with data normalized to β-Actin.

**Table 1.** List of Primary and Secondary Antibodies Used for Western Blotting

Protein Target	Host Species	Concentration	Product Code	Secondary Antibody
Total YAP	Mouse	1:250	sc-101199, (Santa Cruz Biotechnology)	Mouse (m-IgGk BP) conjugated to HRP sc-516102 (Santa Cruz Biotechnology).
pYAPy357	Mouse	1:1000	ab62751 (Abcam, UK)	Mouse (m-IgGk BP) conjugated to HRP sc-516102 (Santa Cruz Biotechnology).
pYAPs127	Rabbit	1:500	#4199 (Cell Signaling Technology)	Goat anti-rabbit IgG-HRP-conjugated secondary antibody 7074 (Cell Signaling Technology)
CTGF	Mouse	1:1000	sc-101558 (Santa Cruz Biotechnology)	Mouse (m-IgGk BP) conjugated to HRP sc-516102 (Santa Cruz Biotechnology).
β-Actin	Rabbit	1:1000	ab8227 (Abcam, UK)	Goat anti-rabbit IgG-HRP–conjugated secondary antibody 7074 (Cell Signaling Technology)

HRP, horseradish peroxide.

## Real-Time Reverse Transcription PCR

Following completion of cell culture in their respective conditions, culture medium was removed and TRIzol Reagent Solution (Life Technologies, Carlsbad, CA, USA) was added to the flasks/wells. Samples were transferred to a 1.5 mL Eppendorf tube and placed on ice. Chloroform 200 of was added per 1 mL of TRIzol, and the sample was inverted to ensure mixing. Samples were incubated on ice for five minutes and then spun in a centrifuge at 12,000 *g* for 15 minutes at 4°C to allow phase separation. The upper, clear aqueous phase containing the RNA was carefully removed and placed into a new Eppendorf tube on ice. Isopropanol 500 µL was added to the aqueous phase and kept on ice for five minutes, and then spun in a centrifuge at 12,000 *g* for eight minutes at 4°C. The supernatant was removed and discarded, and the pellet was washed with 1 mL 75% ethanol and spun in a centrifuge at 7500*g* for five minutes at 4°C. The supernatant was removed, and the pellet was allowed to air dry. The RNA pellet was resuspended in diethylpyrocarbonate-treated water and stored at −80°C for subsequent conversion to complimentary DNA.

RNA was reverse transcribed to complimentary DNA as follows; A 0.2 mL PCR Eppendorf was filled with 11 µL diethylpyrocarbonate water (Sigma-Aldrich Corp.), 1 µL oligo dT (Sigma-Aldrich Corp.), 1 µL deoxynucleotides (Sigma-Aldrich Corp.), 2 µL of 10× AMV reverse transcriptase buffer (Sigma-Aldrich Corp.), and 4 µL RNA. This was heated to 70°C for 10 minutes and then left on ice for two minutes. AMV reverse transcriptase 0.5 µL was added and the tube transferred to the thermocycler. The cycle was 45°C for 90 minutes, 90°C for two minutes, and then cooled to 4°C and stored at −20°C.

RT-qPCR was performed using 18S as a housekeeping gene to normalize Ct values. A standard qPCR cycle was used as follows: denaturation at 95°C for five minutes, denaturation at 95°C for 10 seconds, annealing at 60°C for 20 seconds, and elongation at 72°C for 20 seconds. This was repeated from the second denaturation step for a total of 45 cycles, followed by a melt curve program of 95°C for five seconds and 65°C for one minute. Fold change in gene expression was assessed using the 2^ΔΔCt^ equation. Primer sequences for genes used are shown in Table 2.

**Table 2.** Primer Sequences for Quantitative Real-Time RT-PCR

Gene Name	Forward	Reverse
18S	*5* *ʹ* *-GTAACCCGTTGAACCCCATT*	*5* *ʹ* *-CCATCCAATCGGTAGTAGCC*
YAP1	*5* *ʹ* *-CCTCTTCCTGATGGATGGGAAC*	*5* *ʹ* *-TATTCCGCATTGCCTGCCG*
COL1A1	*5* *ʹ* *-TTCTGTACGCAGGTGATTGG*	*5* *ʹ* *- CATGTTCAGCTTTGTGGACC*
α-SMA	*5* *ʹ* *-AAAGCTTCCCAGACTTCCGC*	*5* *ʹ* *- TTCTTGGGCCTTGATGCGAA*

### Immunofluorescence

NLC cells were seeded on soft (4 kPa) and stiff (100 kPa) collagen-1, polyacrylamide gels overlying detachable coverslips, cultured for 24 hours, serum starved overnight, before being treated with DMSO (vehicle only) or DMSO/VP (2 µM) for a further 24 hours (experimental triplicate). They were then fixed in 100% ice-cold methanol for five minutes and blocked in PBS containing 0.1% Triton X-100 and 5% normal goat serum (ThermoFisher Scientific) for 1 hour at room temperature. They were subsequently probed for total-YAP (sc-101199, 1:50 dilution in blocking buffer) overnight at 4°C. Coverslips were washed three times in PBS. Species-specific secondary antibodies (A10521) and F-actin (Phalloidin A12379; ThermoFisher Scientific) were diluted 1:500 and 1:3000 in PBS respectively, and incubated for two hours at room temperature and protected from light. Coverslips were washed again, and for the third wash we used 4ʹ,6- diamidino-2-phenylindole at a 1:5000 dilution for nuclear localization. Coverslips were washed a final time and were mounted to slides using aqua-polymount (Polysciences, Warrington, PA, USA). Digital images were captured using a confocal microscope (Zeiss LSM 710; Zeiss, Oberkochen, Germany) and processed using imaging software ZEN Lite.

### Cell Proliferation Assay

Cell proliferation MTS assays were used to compare the proliferation rate in LC cells from glaucomatous patients and age-matched normal donors. Freshly thawed GLC and NLC cells from the same passage were seeded onto “CellTiter 96” 96-well flat-bottom plates (Promega Corporation, Madison, WI, USA) at a density of 10^3^ cells per well in a final volume of 100 µL/well. Cell proliferation was measured at different time points (72, 96, 120 hours) using the MTS colorimetric cell counting assay according to the manufacturer's protocol (Promega Corporation). MTS is bioreduced by cells into a colored formazan product that reduces absorbance at 490 nm. After incubation, 20 µL of the CellTiter 96 AQueous One Solution Reagent (Promega Corporation) was added to each well, and the plates were incubated in a humidified 5% CO_2_ atmosphere for one hour. The supernatant was removed, and 150 µL/well of DMSO was added to the plates to solubilize the formazan salt crystals. Plates were incubated for 10 minutes at room temperature. Solubilized formazan products were quantified using a Molecular Devices, Spectra Max multiple microplate reader (Molecular Devices, San Jose, CA, USA) at a wavelength of 490 nm.

To ascertain the effect of substrate stiffness on proliferation rates, we repeated the above experiment using NLC cells and a 96 well plate of incrementally increased substrate stiffness values; 0.2/0.5/1/2/4/8/12/25/50 kPa and tissue culture plastic (approximately 10^6^ kPa) (High Throughput Screen, Matrigen Products; Cell Guidance Systems) at a fixed timepoint of 96 hours. Additionally, we studied the effect of VP on proliferation using vehicle only (DMSO) or VP/DMSO at a concentration of 2 and 4 µM VP for a period of 24 hours after a 24-hour period for cell attachment and further overnight serum starvation at stiffness values of 4 kPa, 50 kPa, and tissue culture plastic.

### Statistical Analysis

Data were expressed as means of independent experiments ± SEM. Statistical significance was analyzed by Student's *t*-test (two-tailed; paired or unpaired as appropriate) for comparison between two groups, and by one-way ANOVA with Tukey-Kramer post-test for multiple comparisons. *P* < 0.05 was taken as the level of significance (*P* < 0.05). Calculations were performed using the Origin 7.0 software (OriginLab, Northampton, MA, USA).

## Results

### YAP Expression is Elevated in LC Cells from Glaucomatous Patients

**Figure 1.**
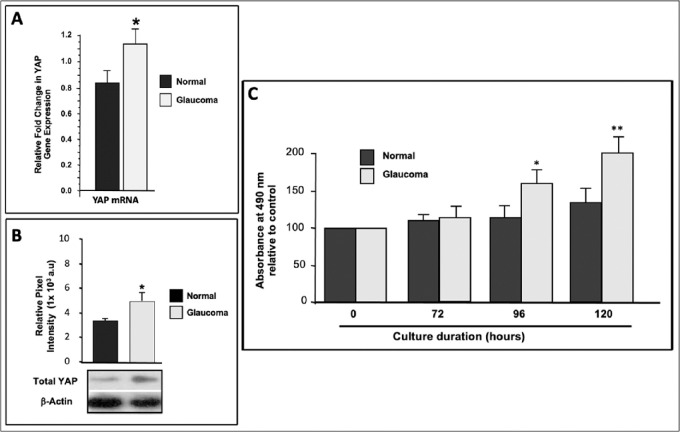
Comparison of LC cells from optic nerve heads of normal and glaucomatous age-matched patients with respect to YAP levels and proliferation rates. (**A**) Quantitative real-time PCR demonstrating YAP gene expression levels in LC cells from normal versus glaucomatous patients, normalized to the housekeeping gene 18 s. There is a significant increase in YAP mRNA in glaucomatous (1.138 ± 0.116) compared to normal LC cells (0.849 ± 0.09) (n = 3) (*P* < 0.05). (**B**) Western blot for total-YAP in both normal and glaucomatous donor LC cell lysates normalized to beta actin shows a significant increase in YAP expression by glaucomatous cells (3.42 ± 0.18 to 5.1 ± 0.63 (10^3 ^a.u) (*P* < 0.05). (**C**) Proliferation rates of LC cells in normal compared to glaucomatous donors (n = 3) are similar at 72 hours; (108.7 ± 9.5 vs. 112.4 ± 17.6), but significantly lower at 96 hours; (113.2 ± 17.5 vs. 159.1 ± 18.8) (*P* < 0.05) and 120 hours (132.6 ± 19.7 vs. 201.2 ± 22.4) (*P* < 0.02).

YAP mRNA levels were compared via RT-PCR in GLC cells and age-matched NLC cells and the difference was expressed as a fold change relative to normalized GAPDH. YAP expression was significantly elevated, by approximately 3 fold, in GLC versus NLC cells, 1.138 ± 0.116 versus 0.849 ± 0.095 (*P* < 0.05; Fig. 1A). Furthermore, Western blotting for YAP protein corresponded to RNA expression levels, with LC cells from glaucomatous patients having higher levels than age matched controls (5.1 ± 0.63 vs. 3.42 ± 0.18 × 10^3^ a.u., *P* < 0.05; Fig. 1B).

### LC Cells from Glaucomatous Donors Proliferate at a Higher Rate

Because YAP is a key regulator of cell proliferation whereby its overexpression enhances proliferation, we examined LC cell proliferation rates in GLC and age-matched NLC cells using MTS assays (n = 3). Accordingly, we found significantly increased proliferation of GLC versus NLC cells at both 96 hours (113.2 ± 17.5 vs. 159.1 ± 18.8, *P* < 0.05) and 120 hours (132.6 ± 19.7 vs. 201.2 ± 22.4, *P* < 0.05; Fig. 1C).

### YAP Regulation in NLC Cells is Highly Mechanosensitive and Inhibited by Verteporfin

**Figure 2.**
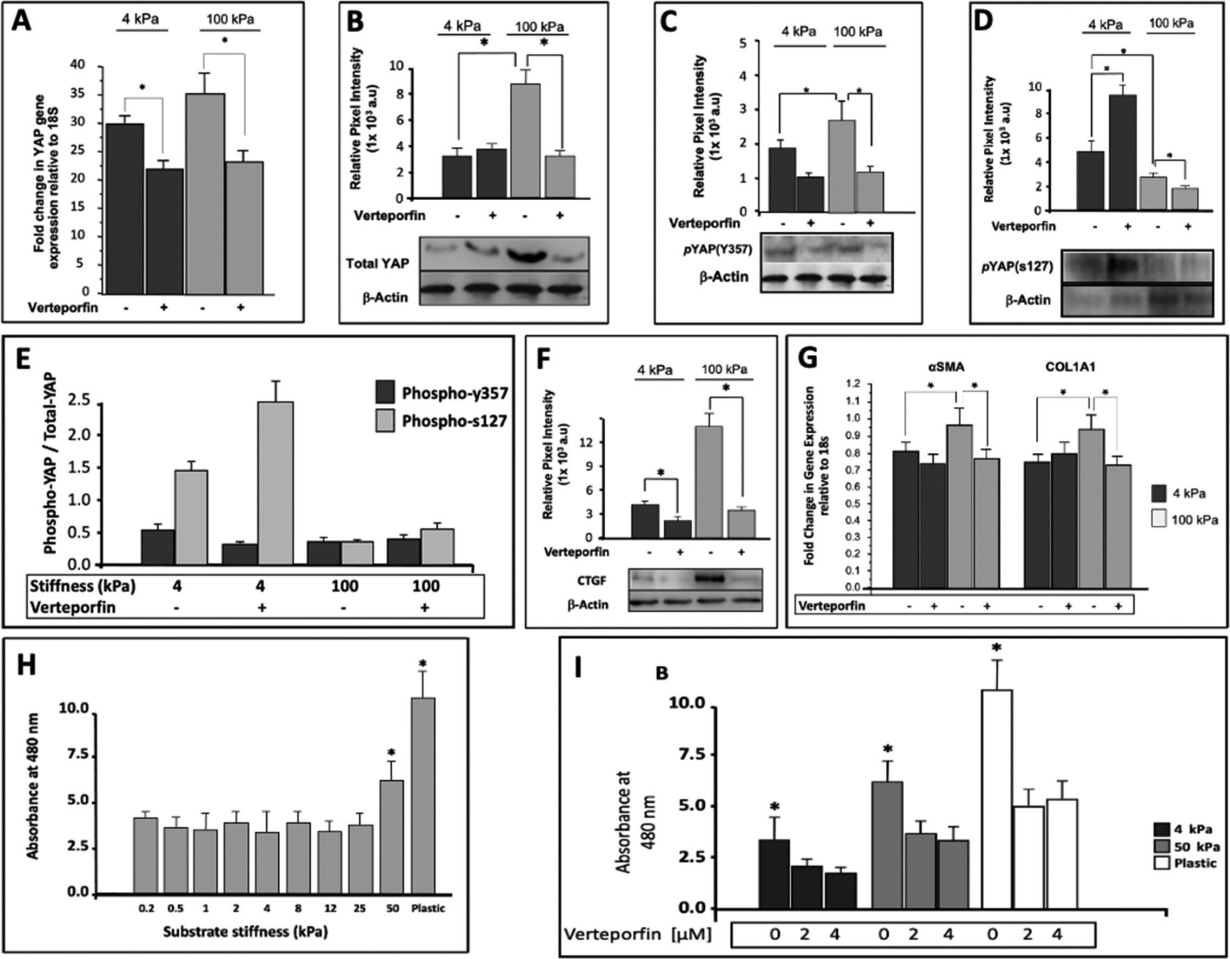
YAP regulation in NLC cells and subsequent cellular proliferation is mechanosensitive and can be inhibited via VP treatment. (**A**) Quantitative real time PCR shows increased YAP gene expression levels when cells are cultured on a stiffer substrate for 24 hours (30.1 ± 1. 22 [4 kPa] vs. 35.8 ± 4.17 [100 kPa]) (values expressed as fold change normalized to housekeeping gene 18 s) (n = 3; *P* ≤ 0.05). When treated with VP (2 µM) for 24 hours, there is reduced YAP gene expression on both soft (30.1 ± 1. 22 untreated, vs. 22.4 ± 2.1 treated) and stiff (35.8 ± 4.17 untreated, vs. 22.71 ± 2.23 treated; n = 3; *P* < 0.05) compared to vehicle control (DMSO). (**B**) Western blot demonstrating differential total-YAP protein levels between soft (4 kPa) and stiff (100 kPa) substrates in the presence (+) and absence (−) of VP (2 µM) for 24 hours. Total-YAP increases from soft to stiff substrates (3.26 ± 0.58 [×10^3^ a.u]; 4 kPa vs. 8.63 ± 1.19 [×10^3^ a.u; 100 kPa; *P* < 0.05]). Verteporfin treatment significantly reduces the stiffness-induced total-YAP expression levels at a pathomimetic stiffness of 100 kPa (8.63 ± 1.19 [×10^3^ a.u; untreated] vs. 3.12 ± 0.49 [×10^3^ a.u; treated; *P* < 0.05]). (**C**) Western blot demonstrates differential YAP phosphorylation at tyrosine 357 (pYAP[y357]) between soft (4 kPa) and stiff (100 kPa) substrates in the presence (+) and absence (−) of VP (2 µM) for 24 hours. The pYAP(y357) increased from soft to stiff substrates (1.88 ± 0.51 [×10^3^ a.u; 4 kPa] vs. 2.76 ± 0.97 [100 kPa]; *P* < 0.05). Verteporfin treatment reduced pYAP(y357) levels (1.88 ± 0.51 [×10^3^ a.u]; vs. 1.07 ± 0.22 [4 kPa] and 2.76 ± 0.97 vs. 1.21 ± 0.24 [100 kPa;] *P* < 0.05). (**D**) Western blot for pYAP(s127) shows a reduction from soft to stiff substrates (4.82 ± 0.91 [×10^3^ a.u; 4 kPa] vs. 2.86 ± 0.34 [100 kPa]; *P* < 0.05). Verteporfin treatment increased pYAP(s127) levels on soft substrates (9.53 ± 1.08 [×10^3^ a.u]) but decreased pYAP(s127) levels on stiff substrates (2.86 ± 0.34 vs 1.84 ± 0.27; *P* < 0.05). (**E**) Analysis of Western blots for pYAPs127 and pYAPy357 relative to total-YAP shows that pYAPs127 is increased in cells on soft substrates that were untreated compared to those treated (1.47 ± 0.113 vs. 2.56 ± 0.323) but decreased in untreated cells on soft versus stiff substrates (1.47 ± 0.113 vs. 0.33 ± 0.037). VP treatment increased pYAPs127 in cells on stiff substrates (0.33 ± 0.037 vs. 0.58 ± 0.071). However, pYAPy357 in cells was reduced after treatment with VP on soft substrates (0.57 ± 0.064 vs. 0.28 ± 0.034) but no significant change with treatment on stiff substrates (0.31 ± 0.043 vs. 0.38 ± 0.055). (**F**) Western blot demonstrating differential expression by cells of CTGF protein levels, a direct downstream YAP transcriptional target, between soft (4 kPa) and stiff (100 kPa) substrates in the presence (+) and absence (−) of VP (2 µM) for 24 hours. CTGF is increased on stiff substrates (4.34 ± 0.63 vs. 14.22 ± 1.53 [×10^3^ a.u]; *P* < 0.05). Verteporfin treatment reduced CTGF on both soft (4.34 ± 0.63 vs. 2.93 ± 0.27) and stiff substrates (14.22 ± 1.53 vs. 3.41 ± 0.68 [×10^3^ a.u]; *P* < 0.05). (**G**) Shown is the differential expression of profibrotic markers α-SMA and COL1A1, between soft (4 kPa) and stiff (100 kPa) substrates in the presence (+) and absence (−) of VP (2 µM) for 24 hours. There is an increase from soft to stiff substrates, (α-SMA, on 4 kPa = 0.81 ± 0.06 fold change; α-SMA, on 100 kPa = 0.96 ± 0.09 fold change; COL1A1 on 4 kPa = 0.75 ± 0.04 fold change; COL1A1 on 100 kPa = 0.95 ± 0.08 fold change). VP treatment led to a reduction in both markers despite culture on stiff substrates; α-SMA: 4 kPa = 0.81 ± 0.06 versus 0.74 ± 0.05; 100 kPa = 0.96 ± 0.09 vs. 0.77 ± 0.05 (*P* < 0.05); COL1A1: 4 kPa = 0.75 ± 0.04 versus 0.81 ± 0.05; 100 kPa = 0.95 ± 0.08 versus 0.74 ± 0.04 (*P* < 0.05). (**H**) Proliferation rates of nonglaucomatous LC cells across a range of incrementally stiffer substrates, as measured with MTS assays, demonstrated a significant increase in proliferation at 50 kPa (*P* < 0.05) and tissue culture plastic (*P* < 0.05). (**I**) Verteporfin treatment significantly reduced proliferation as measured via MTS assays, across stiffness values of 4 kPa, 50 kPa, and tissue culture plastic, at both 2 µM and 4 µM (*P* < 0.05), with greater effect at stiffer values, although it did not act in a dose-dependent manner.

Real-time PCR displayed enhanced YAP gene expression in NLC cells when grown on a stiffer (100 kPa) substrate compared to soft (4 kPa) substrates, showing 30.1 ± 1. 22 versus 35.8 ± 4.17 fold change (n = 3, *P* ≤ 0.05, Fig. 2A). NLC cells cultured on soft and stiff substrates were then subjected to vehicle only (DMSO) or VP (2 µM) for 24 hours. VP treatment reduced YAP gene expression on both soft (30.1 ± 1. 22 untreated, vs. 22.4 ± 2.1 treated) and stiff substrates (35.8 ± 4.17 untreated, vs. 22.71 ± 2.23 treated, n = 3, *P* < 0.05).

Not only is total YAP significantly elevated on stiff substrates, (3.26 ± 0.58 vs. 8.63 ± 1.19 × 10^3^ a.u, *P* < 0.05; Fig. 2B), but additionally the mechanosensitive Src-mediated phosphorylation of YAP at tyrosine 357 (pYAP[y357]) is increased on stiffer substrates from 1.88 ± 0.51 to 2.76 ± 0.97 × 10^3^ a.u, *P* < 0.05; Fig. 2C). In contrast, Hippo-mediated cytosolic pYAPs127 is decreased on stiffened substrates (4.82 ± 0.91 vs. 2.86 ± 0.34 × 10^3^ a.u, *P* < 0.05; Fig. 2D). Western blot analysis of total-YAP protein after VP treatment demonstrated reduced levels at pathomimetic stiffness of 100 kPa (8.63 ± 1.19 vs. 3.12 ± 0.49 × 10^3^ a.u, *P* < 0.05; Fig. 2B), reduced pYAP(y357) (1.88 ± 0.51 vs. 1.07 ± 0.22 × 10^3^ a.u, *P* < 0.05 and 2.76 ± 0.97 vs. 1.21 ± 0.24 × 10^3^ a.u, *P* < 0.05, Fig. 2C). However, VP treatment increased pYAP(s127) levels on soft substrates (4.82 ± 0.91 vs. 9.53 ± 1.08 × 10^3^ a.u) but decreased pYAP(s127) levels on stiff substrates (2.86 ± 0.34 vs. 1.84 ± 0.27, *P* < 0.05).

Although potential differences in primary or secondary antibody concentrations preclude direct comparison of protein levels of phosphorylated YAP y357 to s127 or to total YAP, we compared the relative changes in such levels across different conditions, namely on soft and stiff substrates in the presence and absence of VP (Fig. 2E). YAP phosphorylation at y357 largely mirrors fluctuating levels of total YAP; where it is increased to a similar degree in stiffer environments and reduces in line with total YAP when treated with VP(Fig. 2E). In contrast, Hippo-mediated YAP phosphorylation at s127 seems to change significantly relative to total YAP, with a significant decrease when cultured on stiffer substrates, but also a significant increase when treated with VP, relative to total YAP on both soft and stiff substrates (Fig. 2E).

### Mechanoactivation via Increased Substrate Stiffness Increases Profibrotic Markers of NLC Cell Activation and is Successfully Inhibited by Verteporfin Treatment

Connective tissue growth factor (CTGF) is a transcriptional target gene of YAP.^26^ We therefore used this as a readout for YAP functional activity/transcriptional output in stiffened substrates. We observed an increase in CTGF protein levels stiffer substrates (4.34 ± 0.63 vs. 14.22 ± 1.53 × 10^3^ a.u, *P* < 0.05; Fig. 2F). VP treatment reduced transcriptional target CTGF on both soft (4.34 ± 0.63 vs. 2.93 ± 0.27) and stiff substrates (14.22 ± 1.53 vs. 3.41 ± 0.68 × 10^3^ a.u, *P* < 0.05; Fig. 2F).

In accordance with increased YAP, markers of myofibroblastic transformation and ECM remodeling, such as α-SMA and COL1A1 are upregulated approximately twofold on stiff substrates compared to soft (for α-SMA, 0.81 ± 0.06 vs. 0.96 ± 0.09 and for COL1A1 0.75 ± 0.04 vs. 0.95 ± 0.08, n = 3; *P* < 0.05, Fig. 2G). Despite culture on a stiffer substrate, treatment with VP reduced markers of cellular transformation. The α-SMA was reduced from 0.81 ± 0.06 to 0.74 ± 0.05 fold change on soft and from 0.96 ± 0.09 to 0.77 ± 0.05 fold change on stiff substrates (*P* < 0.05). Similarly, COL1A1 was reduced on stiff substrates from 0.95 ± 0.08 versus 0.74 ± 0.04 (*P* < 0.05; Fig. 2G).

### NLC Cell Proliferation is Increased on Stiff Substrates and Reduced With Verteporfin Treatment Despite Increased Substrate Stiffness

Following up on our observations of increased proliferation in LC cells from glaucomatous donors compared to age-matched nonglaucomatous donors, we tested whether healthy cells cultured in pathomimetic substrates adopt a higher proliferation rate. NLC cells cultured for 96 hours on incrementally increased substrate stiffnesses of 0.2/0.5/1/2/4/8/12/25/50 kPa and tissue culture plastic (approximately 10^6^ kPa), proliferate at significantly higher rates on substrates having a stiffness of 50 kPa (*P* < 0.05) and on tissue culture plastic (*P* < 0.05; Fig. 2H). This increase was not seen in a linear fashion but rather it seemed that proliferation was constant until a threshold value of 50 kPa whereby a significant change incurred.

We then compared the extent of VP inhibition on stiffness-induced proliferation at significantly influential, stiffness values of 4 kPa, 50 kPa and tissue culture plastic. VP treatment at both 2 µM and 4 µM significantly reduced proliferation of cells on substrates at all three stiffnesses tested (*P* < 0.05), although its action did not appear to be dose-dependent. (Fig. 2I)

### Subcellular Localization of YAP is Predominantly Nuclear on Stiff Substrates But This is Abrogated After Treatment With VP

**Figure 3.**
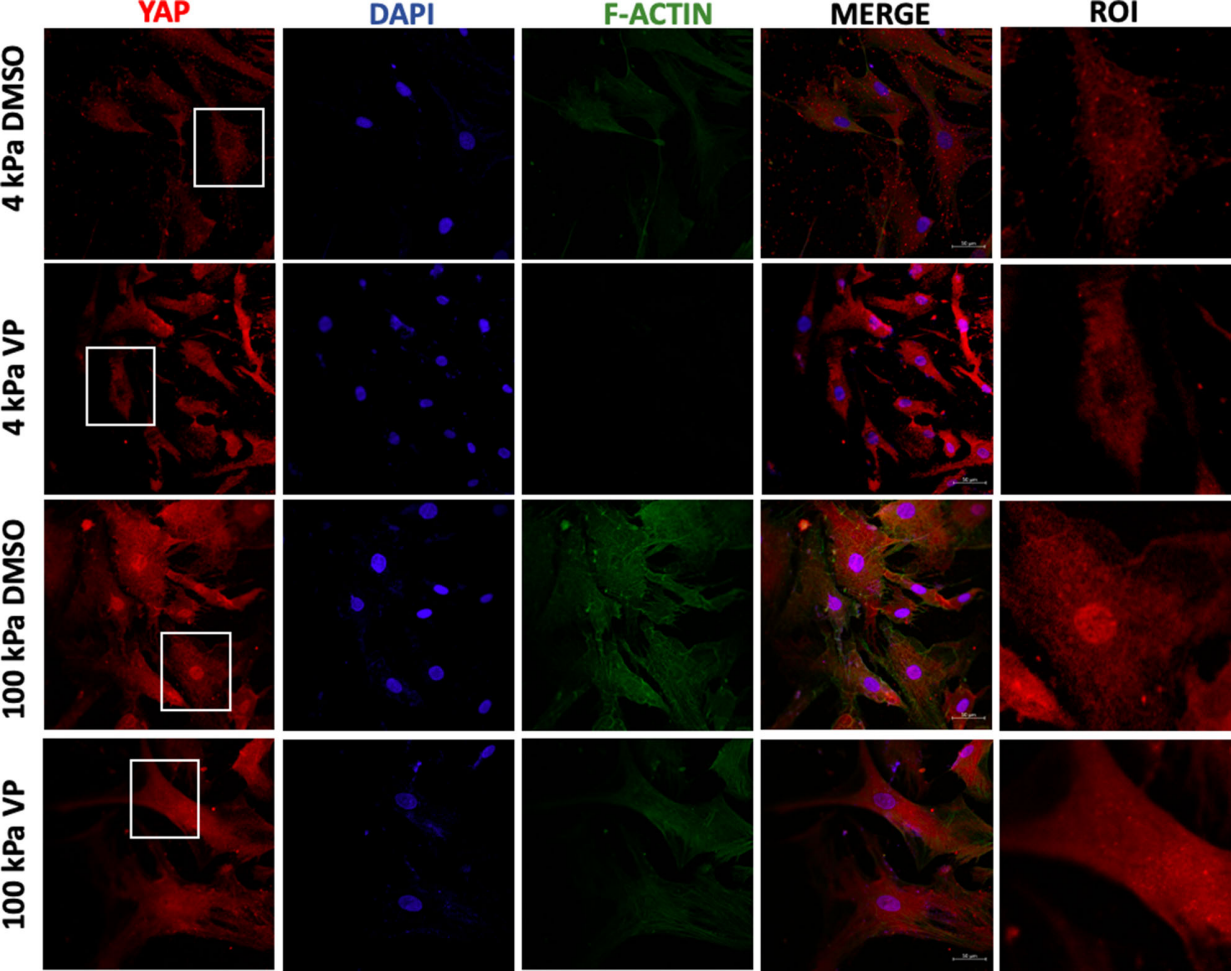
Effect of substrate stiffness and VP treatment on the subcellular localization of YAP. NLC cells were seeded on soft (4 kPa) and stiff (100 kPa) collagen-1, polyacrylamide gels overlying detachable coverslips (Softslip, Matrigen Products; Cell Guidance Systems), cultured for 24 hours, serum starved overnight, and exposed to either vehicle only (DMSO) or VP (2 µM) for an additional 24 hours. Cells were subsequently probed for total-YAP, F-actin and 4ʹ,6- diamidino-2-phenylindole for nuclear localization. Digital images were captured using a confocal microscope (Zeiss LSM 710; Zeiss) using identical settings, and processed using imaging software ZEN Lite. The “region of interest” is a magnified view of the *white box* overlayed on the images to the first column (YAP), to more closely visualize nuclear/cytoplasmic localization of YAP. NLC cultured on 100 kPa consistently showed greater overall fluorescence and nuclear pooling of total-YAP in comparison to culture on 4 kPa. VP treatment seemed to abrogate this nuclear translocation.

Immunofluorescence microscopy was performed on NLC cells cultured on soft and stiff substrates to determine whether substrate stiffness affects the cellular localization (cytoplasmic vs. nuclear) of YAP. Cells on soft substrates show reduced F-actin staining compared to stiff, and YAP protein was localized in the cytoplasm, appearing excluded from the nucleus (Fig. 3). In contrast, there is a marked increase in fluorescence signal of F-actin on stiff substrates, with YAP brightly staining both the nucleus and cytoplasm. Such a phenotype on a stiff substrate was blunted on treatment of LC cells with VP that resulted in decreased nuclear staining. (Fig. 3)

## Discussion

Considering the emerging importance of YAP regulation in promoting fibrosis and the significant fibrotic remodeling underpinning glaucomatous progression at the LC, the role of YAP in LC cells represents a potentially novel therapeutic avenue independent of IOP and warrants further delineation. As shown by the in vitro experiments described herein, we have developed a mechanistic understanding of the activity of YAP in LC cells. We compared LC cells from non-glaucomatous and age-matched glaucomatous eye donors, demonstrating enhanced YAP gene expression and upregulated total-YAP protein in glaucoma. Consistent with the proliferative nature of YAP activation, we showed increased proliferation in GLC cells compared to NLC cells. Furthermore, we interrogated the effect of exposing healthy, non-glaucomatous LC cells to a pathomimetic, stiffened microenvironment, and showed the highly mechanosensitive nature of YAP regulation and corresponding differential phosphorylation at serine and tyrosine sites. Additionally, downstream targets CTGF and myofibroblastic markers α-SMA and COL1A1 were increased. Immunofluorescence microscopy demonstrated how substrate stiffness is a critical determinant of YAP nuclear translocation. Finally, we used a YAP inhibitor, VP, and successfully disrupted stiffness-induced LC cell mechanoactivation. We found that treating NLC cells with VP markedly reduces YAP gene expression and transcriptional induction despite culture on a stiffened substrate. Functionally, there was decreased total YAP, decreased pYAP(y357), and increased pYAP (s127) with VP treatment and a concurrent reduction in CTGF, α-SMA and COL1A1, suggesting reduced transcriptional activity, with visibly reduced nuclear localization by immunofluorescence microscopy (Fig. 3).

Previous work from our group has elucidated differential global and ECM-focused gene expression patterns in LC cells from normal compared to glaucomatous donors.^41^ Several of these differentially expressed genes are involved in YAP regulation. Furthermore, results from a recent multiethnic meta-analysis of several genome-wide association studies identified SNP variants in YAP and the Hippo signaling pathways to be associated with the pathogenesis of POAG.^42^ Our observations presented herein support a mechanosensitive molecular mechanism for YAP dysregulation in glaucomatous LC cells and are consistent with the positive correlations between polymorphisms in the risk loci YAP1 and POAG revealed by genome-wide association studies.^42^ Taken together, they demonstrate compelling evidence for YAP dysregulation as a causative factor in the pathological ECM remodeling seen in glaucoma and suggest stiffening of the cellular microenvironment as a key driver and perpetuator of this maladaptive dysregulation. Consistent with this idea, we found LC cells from glaucomatous patients to have markedly increased YAP expression compared to nonglaucomatous age-matched donors.

In attempting to find a cause for this observation, and to further understand what initially drives YAP upregulation at the onset of glaucoma, we investigated how YAP activity was regulated in healthy NLC cells exposed to a stiffened microenvironment akin to that observed in aging and glaucoma. Age-related ECM remodeling at the LC includes changes to nonenzymatic collagen cross-link pentosidine, total collagen and elastin content, decreased type III to type I collagen ratio, and decreased total sulphated glycosaminoglycans content, all of which infer a stiffer LC with age.^9^^,^^10^ Further research has shown such composite structural changes result in the biochemical basis for a stiffer and less compliant LC with increased age and furthermore that the onset of stiffening seems to correlate with typical POAG onset, at about 40 to 50 years.^10^ These mechanical alterations, as measured by reduced compliance in the glaucomatous LC, have been shown to correlate functionally by extent of visual field damage in glaucomatous individuals.^12^ Together we can assimilate a reasonable suspicion regarding the causative nature of stiffness in the pathogenesis of glaucoma and thus used it to form our hypothesis that substrate stiffness alone could manifest such cellular activation.

Consistent with our hypothesis, we found that mimicking the stiffened “in vivo” glaucomatous LC for healthy nonglaucomatous LC cell culture led to a comparable upregulation of YAP “in vitro” (Fig. 2). Increased nuclear transcriptional activity was observed, as inferred by markedly enhanced nuclear localization of total-YAP on immunofluorescence, increased transcriptionally active pYAP(y357), with reduced pYAP(s127), and confirmed by measurement of its direct transcriptional target, CTGF, and functionally witnessed through increased proliferation. These results are in keeping with the highly mechanosensitive nature of YAP and its established role in tissue homeostasis.^43^ We propose that YAP overexpression is in part responsible for the aging and stiffening LC, but that for glaucomatous individuals, there exists a heightened YAP-mediated response to substrate stiffness, condemning them to a progressive positive feedback loop of ECM deposition and fibrosis.

Linking ECM stiffness to YAP dysregulation, we note that several gene variants implicated in glaucoma such as caveolin 1 and 2 (CAV1,CAV2), lysyl oxidase–like 1 (LOXL1), and myocilin have separately been shown to modulate YAP regulation in a highly mechanosensitive manner, through actin-cytoskeleton–dependent, MST1/2 kinase, and Wnt signaling pathways, respectively.^42^^,^^44^^–^^50^ Furthermore, several mechanosensitive YAP regulators have also been shown to be differentially expressed in the trabecular meshwork, aqueous humor, or optic nerve head of glaucomatous eyes, such as Secreted frizzled-related protein 1 (sfrP1) (a Wnt pathway inhibitor), TGF-β, thrombospondin-1, hypoxia-inducible factor 1α, TNF-α, cell cycle inhibitor P27, LPA, lysophosphatidylcholine, autotaxin, and CTGF.^51^^–^^60^ Moreover, matrix stiffening is thought to dominate the cellular response to soluble cues, because evidence points to the requirement of a mechanically stressed cytoskeleton for effective GPCR/WNT pathway activation through YAP.^32^^,^^61^ The powerful effector role that YAP plays, as the converging end-point of these pathways, makes it an attractive and potentially highly effective therapeutic target.

**Figure 4.**
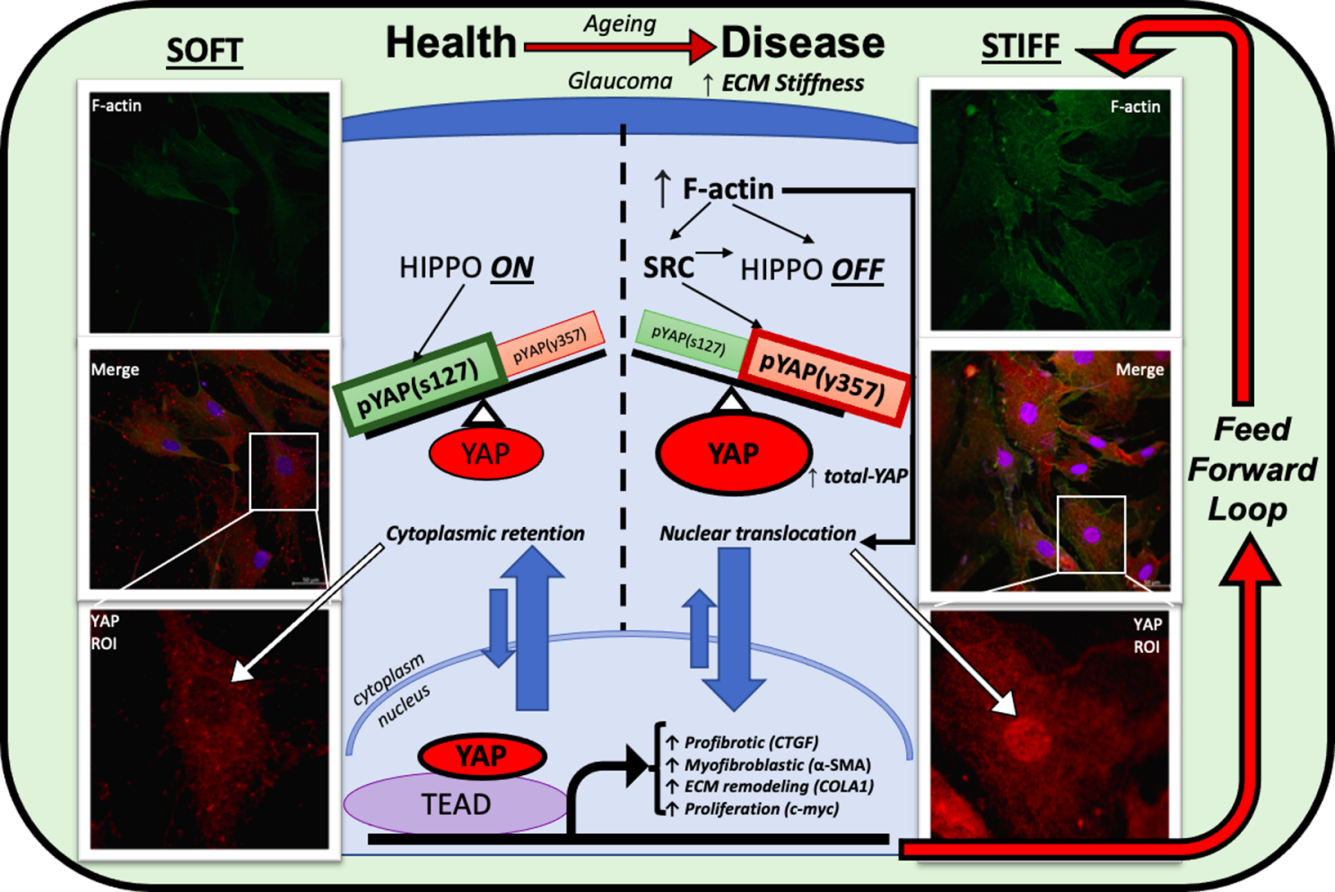
YAP regulation in health and disease. Schematic of healthy LC cells in soft substrate (left) transforming to an activated state (right). The schematic is split in half to juxtapose YAP regulation in health (soft ECM), compared to the right side, which resembles increased ECM stiffness, glaucoma, and YAP dysregulation. We represent the following molecular changes within the schematic; increased F-actin, increased YAP phosphorylation at tyrosine 357, and reduced phosphorylation at serine 127, increased total-YAP, increased nuclear translocation and increased nuclear import (in part via direct F-actin–mediated pore opening) reduced nuclear export, with increased transcriptional activity of YAP/TEAD complex transcriptional targets. This results in increased ECM protein synthesis, increased myofibroblastic markers, increased activating growth factors and increased proliferation. This cycle is self-perpetuating as shown on the right by a “feed forward loop.”

The specific phosphorylation site of YAP is of critical importance in determining its activity as a transcriptional co-activator, but is becoming recognized as increasingly complex. Earlier publications focused largely on the well characterized Hippo-mediated YAP phosphorylation at serine 127, resulting in a “Hippo-on” state, which results in cytosolic retention via binding with 14-3-3 proteins, reduced transcriptional activity due to exclusion from the nucleus, and proteasomal degradation.^62^ Conversely, “Hippo-off” activates YAP via loss of phosphorylation at s127, disruption of its “stable” cytoplasmic retention, and translocation to the nucleus. Although Hippo-signaling is important in the functional fate of YAP, as a determinant of subcellular location, it is not exclusive, and rather SRC-mediated YAP phosphorylation at tyrosine 357 has been shown to independently promote nuclear translocation regardless of serine 127 phosphorylation status, and further that the same SRC pathway negatively regulates the Hippo pathway to a state of “Hippo-off.”^63^ In contrast to previously held theories, several more recent live imaging studies have established that there exists YAP, which is nuclear, yet phosphorylated at serine 127, and that it exists in a state of dynamic fluctuations, revoking prior principles of an oversimplified binary on/off status.^64^^–^^66^ Recent findings suggest that nuclear export from within the nucleus is the greatest determinant of the subcellular location of YAP, rather than reduced nuclear import secondary to cytoplasmic retention via YAP-14-3-3 binding.^64^ Not only is YAP held in the nucleus for longer, (avoiding degradation), but its actual duration of attachment to chromatin is increased when it is phosphorylated at tyrosine 357. Consistent with this concept was our finding that pYAP(y357) was markedly increased on stiffened substrates, total-YAP was predominantly nuclear on stiffened substrates as seen through immunofluorescence, and its transcriptional activity was validated by increased CTGF and cellular proliferation (Figs. 2 and 3). Equally, lower levels of pYAP(s127) relative to total YAP were observed on stiff substrates, and this corresponded to the nuclear translocation witnessed on immunofluorescence and increased transcriptional output (Fig. 2E). We found greater differential expression of pYAP(s127) (via Hippo pathway) relative to total YAP, across soft and stiff substrates, when compared to pYAP(y357), perhaps suggesting that the Hippo pathway is implicated more than the SRC-mediated phosphorylation at tyrosine y357 in the mechanosensitive regulation of YAP. We have illustrated these findings in Figure 4, whereby a “seesaw” balance involving both YAP phosphorylation at serine 127 and tyrosine 357, determines subcellular location, and that there exists constant shuttling in and out of the nucleus, determined by the balance of phosphorylation site (Fig. 4). A further determinant of YAP activity is through a direct mechanical connection between focal adhesions at the cell membrane and the nucleus, mediated through F-actin cytoskeleton.^66^ Exposure to stiffened microenvironment is transmitted to the nucleus, resulting in nuclear flattening, stretching of the nuclear pores, reducing their resistance to molecular transport and increasing YAP nuclear import.^66^ This is consistent with our immunofluorescence findings of NLC cell culture on 4 kPA versus 100 kPA substrates with increased F-actin and nuclear YAP being visible across all cells on stiffened substrates (Fig. 3).

In light of the important role of YAP in cellular proliferation, together with our findings of YAP upregulation in glaucomatous LC cells, we demonstrated increased proliferation of NLC cells on stiffened substrates (Fig. 1C).^31^^,^^67^^–^^69^ The contextual importance of this can be found in recognizing the LC cell as the main effector of ECM remodeling and that it follows that more abundant LC cells will lead to increased collagen and fibronectin synthesis, increased matricellular proteins such as CTGF, and thus lead to a feed-forward cycle of progressive LC stiffening.^70^ We report our findings of LC proliferation with interest given that it appears to suggest a threshold level of substrate stiffness for increased cell proliferation at 50 kPa whereby above this level, intracellular YAP-mediated homeostasis is lost. There is a general consensus that the LC is stiffer in POAG, although estimations of fold value vary and are inferred from ocular structures including trabecular meshwork (TM) tissue stiffness reported by Last et al.,^71^ who suggested a 20-fold increase in stiffness in glaucoma through atomic force microscopy, although more recent studies suggest qualitative rather than quantitative agreement with glaucomatous TM, Schlemm's canal, and peripapillary scleral stiffening, with ranges as low as 2 fold increased stiffness in glaucoma.^72^^–^^74^ Nevertheless, proof of concept regarding stiffness-mediated increased proliferation in NLC cells is achieved herein. The potential for disparity between stiffness values chosen in this study and those sensed by LC cells in vivo is undoubtedly a limitation in the implications of our in vitro findings. In vitro cell cultures, as performed herein, cannot capture the inherent complexity of an in vivo LC cell's microenvironment, and we are reluctant to suggest that our findings do so. Furthermore, YAP activity may differ with age, race, and other variables within a population, and these differences may not be reflected in our limited sample size.

Given that there are several formally discreet mechanisms for YAP regulation (F-actin cytoskeleton, SRC-mediated y357 phosphorylation, Hippo-mediated s127 phosphorylation, and 14-3-3 YAP regulation), the inhibition of the convergent endpoint of YAP-TEAD binding represents an attractive and potentially highly effective prospect. In this study, we used VP without light activation. Verteporfin is a small-molecule benzoporphyrin derivative, commonly used as a photosensitizer for photodynamic therapy, with Food and Drug Administration approval for treatment of neovascular age-related macular degeneration, presumed ocular histoplasmosis, and pathological myopia, and off-label use, with laser, for central serous retinopathy.^75^^,^^76^ In the realms of cancer biology and the study of chronic fibrotic diseases, VP without light activation has recently become recognized as a potent YAP inhibitor, blocking downstream transcriptional targets, and although its exact inhibitory mechanisms haven't been fully elucidated, its ability to disrupt the nuclear YAP/TEAD complex has been well characterized.^77^ In addition, there are several other mechanisms of VP action recently identified which operate independent of YAP/TEAD complex formation, including modulating the PI3K, Ras, mTOR, and Wnt signal pathways.^78^ Moreover, there has been speculation regarding further mechanisms of action of VP other than disruption of YAP-TEAD complex formation, and one concept gaining traction is the upregulation of protein 14-3-3, which would explain the reduced total-YAP (as it undergoes subsequent proteasomal degradation when bound to 14-3-3) and increased pYAP(s127) (Fig. 2).^79^

Exponential research output within the realm of cancer biology has demonstrated successful YAP inhibition with VP in several cell lines. VP treatment has demonstrated decreased cell growth, proliferation, migration, and invasion, with reported molecular mechanisms observed including reduced YAP, vimentin, STAT3, FAK, COX2, SOX2, TEAD, KRAS, mTOR, p-mTOR, ROCK2, MEK, p-MEK, p-ERK, GLUT1, Cyclin-D1, c-myc, Survivin, VEGFA, MMP2, alpha-SMA, IL-11, IL-6, FOXM1, CTGF, CYR61, bcl-2, and increased pYAP(s127), BAX, cleaved PARP, cleaved Caspase 3 and 9.^78^ Translating in vitro results to an in vivo setting is of course challenging, but several in vivo studies determining the antitumorigenic properties of VP have been very encouraging.^78^

Several recent reports have outlined YAP inhibition via VP without light activation in cells of ocular origin, namely TM cells and conjunctival fibroblasts. The first of such studies was by Chen et al.^80^ who examined human TM cells in vitro through culture on collagen gels and used VP without light activation to establish a means for YAP inhibition. They reported that after 24 hours treatment, VP abolishes TM cell–mediated collagen gel contraction in a dose-dependent manner. Additionally, it attenuates expression of YAP and CTGF (a direct YAP target gene) in a dose-dependent manner, and last, it has no significant cytotoxicity below 2 µM. Yemanyi et al.^81^ similarly used hTM cells to uncover the crosstalk between the LPA, Interleukin 6 (IL-6), YAP/TAZ, and Signal transducer and activator of transcription 3. LPA or IL6 trans-signaling were shown to activate YAP, TAZ, and STAT3 overexpressed key components of the actomyosin machinery, in conjunction with increased expression of specific receptors and ligands, α-SMA, and fibrotic ECM genes/proteins. VP abrogated LPA or IL6 trans-signaling–mediated activation of YAP, TAZ, and STAT3. Focusing on conjunctival fibroblasts, Futakuchi et al probed the relationship between TGF-β2 and YAP.^82^ They witnessed YAP activation of SMAD2/3, leading to upregulation of profibrotic genes including α-SMA, fibronectin, collagen I, and collagen IV.^82^ VP was successful at attenuating the observed cellular myofibroblastic activation and this was further confirmed functionally by collagen gel contraction assays. Given the similar pathophysiology of the TM and LC, and furthermore by the potential use for VP treatment in conjunctival cells after filtration surgery, VP presents an exciting prospect for further testing in glaucoma.

Our in vitro findings presented herein implicate dysregulated YAP activity at the lamina cribrosa in the pathogenesis of glaucoma. In confirming the mechanosensitive nature of this dysregulation, and its potential inhibition with VP, we highlight the therapeutic potential in repurposing VP without light activation and propose it to be a worthy candidate for further research.
